# Sealpox Virus in Marine Mammal Rehabilitation Facilities, North America, 2007–2009

**DOI:** 10.3201/eid1712.101945

**Published:** 2011-12

**Authors:** Amira A. Roess, Rebecca S. Levine, Laura Barth, Benjamin P. Monroe, Darin S. Carroll, Inger K. Damon, Mary G. Reynolds

**Affiliations:** Centers for Disease Control and Prevention, Atlanta, Georgia, USA

**Keywords:** sea lion, sea lion poxvirus, pinniped, Parapoxvirus, sealpox, sealpox virus, seal lion pox virus, marine mammal rehabilitation, virus

## Abstract

Sealpox, a zoonotic disease affecting pinnipeds (seals and sea lions), can occur among captive and convalescing animals. We surveyed 1 worker each from 11 marine mammal centers and interviewed 31 other marine mammal workers to ascertain their knowledge of and experience with sealpox virus and to identify factors associated with sealpox virus outbreaks among pinnipeds in marine rehabilitation facilities. Demographic and health data were obtained for 1,423 pinnipeds at the 11 facilities. Among the 23 animals in which sealpox was clinically diagnosed, 4 arrived at the facility ill, 11 became ill <5 weeks after arrival, and 2 became ill >5 weeks after arrival; the timing of illness onset was unknown for 6 animals. Most infections occurred in pinnipeds <1 year of age. Nine affected animals were malnourished; 4 had additional illnesses. Sealpox had also occurred among workers at 2 facilities. Sealpox is a noteworthy zoonosis of rehabilitating convalescing pinnipeds; workplace education can help to minimize risks for human infection.

## Abstract 2

Risks for human infection may be appreciable and can be reduced by workplace education.

Sealpox is a zoonotic disease of seals and sea lions (pinnipeds) and can be a complication of animals undergoing rehabilitation (*1**–**4*). The virus has been confirmed in free-ranging pinnipeds in the northern and southern Atlantic and Pacific Oceans (*5**,**6*), and infections have been observed in animals off the coast of Queen Maud’s Land, Antarctica (*7*). Sea lionpox virus is taxonomically and genetically distinct from other sealpox viruses found in Pacific Ocean pinnipeds (*8*); however, for convenience, hereafter we will refer to sea lionpox virus as sealpox virus.

Eight pinniped species are known to be susceptible to infection with sealpox viruses: *Halichoerus grypus* (gray seals), *Phoca vitulina* (harbor seals), *P. groenlandica* (harp seals), *Callorhinus ursinus* (northern fur seals), *Mirounga angustirostris* (northern elephant seals), *Zalophus californianus* (California sea lions), *Eumetopias jubatus* (steller sea lions), and *Otaria flavescens* (South American sea lions) (*9**–**13*). Many of the reported infections have been in young animals brought into rehabilitation environments (*1**,**11*), but infections have also been observed among animals in colonies undergoing exogenous stress (pollution, food scarcity, other underlying infection) and, on occasion, in otherwise seemingly healthy adults. Sealpox virus can spread easily among confined animals and can increase the costs and length of rehabilitation. Studies conducted in pinniped rehabilitation centers have suggested that underlying health conditions and a history of veterinary care are risk factors for sealpox virus infection among California sea lions (*1**,**14*).

Animals infected with sealpox virus typically show development of firm skin nodules (1–3 cm) on the head, neck, and thorax. Solitary clusters of nodules may also be found on the abdomen, flippers, and mucosa or oral cavity (*6**,**13*). The nodules frequently are inflamed or necrotic but usually heal spontaneously within a few weeks, leaving a slightly raised, gray, furless scar. Illness levels can be substantial, but death rates generally are low except among juveniles, for which infections can interfere with feeding (*1**,**2**,**14*). The transmission dynamics of sealpox virus have not been thoroughly investigated, but virus transmission is thought to occur directly by skin-to-skin contact.

Sealpox viruses are tentatively classified in the genus *Parapoxvirus* (*15*), which comprises multiple species of virus that can infect humans. Other members of this genus—namely, orf virus, pseudocowpox virus, and bovine papular stomatitis virus—commonly infect domestic ruminants and pose a minor occupational hazard to producers of sheep, goat, and cattle. The typical manifestations of infection in humans with any of these agents, including sealpox virus, are similar, usually culminating in a single nodular lesion with a diameter of ≈3 mm–1 cm. The nodule is often painful and evolves slowly over the course of several weeks. Infected persons may also briefly experience systemic signs and symptoms (fever, myalgia, fatigue) during the initial stages of lesion formation.

Sealpox virus is likely transmitted to humans when broken skin comes into contact with virus shed from lesions (skin or oral) on infected pinnipeds. Two epidemiologically linked and 1 molecularly confirmed case of sealpox virus infection in marine mammal handlers have been reported (*16**,**17*). In each case, the handler reported cutting himself while handling pinnipeds that had lesions indicative of sealpox virus infection. Although few sealpox virus infections in humans have been reported, and risks to marine mammal workers remain undefined, data indicate that ≈50% of marine mammal handlers have been injured by a marine mammal (*18**,**19*). The prevalence of human sealpox virus infections may therefore be underestimated.

Since the Marine Mammal Protection Act was enacted in 1972, researchers have documented sizable increases in the abundance of animals of several pinniped species in North America (*20**,**21*). Moreover, the number of live-stranded pinnipeds brought to rehabilitation facilities in the United States since the early 1970s has steadily increased over an order of magnitude and now represents hundreds of animals each year (*22*). Thus, compared with 40 years ago, today more opportunities exist for encounters between humans and convalescing pinnipeds.

To better understand the risks for sealpox virus infection in humans, we conducted a study of marine mammal workers at 11 marine mammal centers with rehabilitation capacity for species in North American waters; the objectives of the study were to ascertain the workers’ knowledge of and experience with sealpox virus and to identify factors associated with sealpox virus outbreaks among pinnipeds in rehabilitation centers. In addition, we also performed a survey of 31 marine mammal workers attending a professional conference to learn about their knowledge of and experience with sealpox infection in humans and animals.

## Methods

### Study Population

During 2007–2009, we contacted 25 marine mammal facilities in North America by phone or email to determine whether they met our inclusion criteria and were willing to participate in the survey. We invited facilities to participate that receive wild pinnipeds for research or rehabilitation purposes. Only facilities that had housed >1 pinnipeds during the previous year were asked to participate. Of the 25 marine mammal facilities contacted, 11 met the criteria and agreed to participate. In addition, we asked marine mammal workers attending a professional conference to fill out a separate study survey; 31 agreed to participate.

### Study Design

We asked each of the 11 facilities that met the inclusion criteria to complete a questionnaire. From each facility, 1 person with knowledge of the center (the facility informant) provided the following information about pinnipeds maintained at the facility during the past 12 months: number and species of animals; demographic and health data; and information pertaining to quarantine practices, animal housing, and medical monitoring. We obtained written consent and sent surveys to participants by email or fax. Surveys were designed by using LiveCycle Enterprise Suite 2.5 software (Adobe, San Jose, CA, USA). Completed surveys were returned to us by mail, fax, or email.

### Statistical Analyses

Data were entered in a spreadsheet and analyzed by using IBM SPSS Statistics 17.0 (www-01.ibm.com/software/analytics/spss). Frequencies and proportions were calculated for the following categorical variables: species, facility location, demographics, disinfectants used for cleaning cages, and housing characteristics. Pinnipeds were divided into 2 age groups, 1 comprising those <1 year of age (pups) and the other comprising those >1 year of age (adults). Free-text responses were coded into categorical variables when appropriate. Odds ratios and 95% confidence intervals were calculated for potential risk factors (age, location, sex) associated with animals who had sealpox cases, where appropriate.

## Results

Of the 11 marine mammal facilities surveyed, 5 were located on the eastern (Atlantic) coast and 6 were located on the western (Pacific) coast of North America (Table 1). Survey informants who represented these facilities were staff veterinarians or veterinary technicians (55%), self-described facility directors (18%), or researchers and rehabilitation trainers (27%). Survey informants provided demographic and health information for 1,423 pinnipeds, representing 14 species (Table 1). Of these pinnipeds, 47% were California sea lions and 27% were harbor seals, and 84% of the animals were housed in facilities located along the Pacific coast (Table 1). Five species were represented on both coasts. Fifty-five percent of pinnipeds described were male, and 83% were <1 year of age.

**Table 1:** Characteristics and infection status for 1,423 pinnipeds at 11 marine mammal centers in the 12 months before survey of marine mammal centers, North America, 2007–2009

Species, age group, and residential status	No. animals						
	Sex			Facility location *		With sealpox†, n = 23	Total, N = 1,423
	M, n = 780‡	F, n = 643§		Pacific coast, n = 1,198¶	Atlantic coast, n = 227#		
Animal (species)							
California sea lion (Zalophus californianus)	365	301		662	4	8	666
Stellar sea lion (Eumetopias jubatus)	15	19		25	9	0	34
Northern fur seal (Callorhinus ursinus)	26	15		38	3	0	41
Northern elephant seal (Mirounga angustirostris)	101	72		173	0	1	173
Ribbon seal (Histriophoca fasciata)	1	0		1	0	0	1
Hooded seal (Cystophora cristata)	3	1		0	4	0	4
Gray seal (Halichoerus grypus)	45	18		0	63	3	63
Harp seal (Phoca groenlandica)	17	18		0	35	0	35
Harbor seal (P. vitulina)	198	191		290	99	11	389
Ringed seal (P. hispida)	1	1		2	2	0	2
Spotted seal (P. largha)	2	0		2	0	0	2
Guadalupe fur seal (Arctocephalus gazella)	2	2		4	0	0	4
Bearded seal (Erignathus barbatus)	4	4		0	8	0	8
South American sea lion (Otaria flavescens)	0	1		1	0	0	1
Age group							
Pup, <1 y of age	725	455		969	211	20	1,180
Adult	55	189		229	14	3	243
Residential status**							
Rescue/rehabilitation	661	626		1,071	216	23	1,287
Resident/other	119	17		127	9	0	136

*A total of 6 and 5 facilities each were located on the Pacific and Atlantic coasts, respectively. †≈30 animals were reported to have sealpox, but information regarding species type, age group, and sex was available for only 23. Thus, only these 23 were included in the analysis. Of the 23 ill animals, 5 had laboratory-confirmed sealpox virus infection. ‡Fifteen of 780 animals were ill. §Eight of 643 animals were ill. ¶Fourteen of 1,198 animals were ill. #Nine of 225 animals were ill. **Rescue/rehabilitation refers to pinnipeds that were brought in from the wild for rehabilitation; Resident/other refers to animals brought in for purposes other than rehabilitation.

Informants were asked to recall the approximate numbers of animals in which sealpox was clinically diagnosed during the previous year. If informants provided a range of case counts, the lower number was selected. For the 12-month period, an estimated 30 (2%) animals at the 11 facilities had sealpox; 25 diagnoses were presumptive (based on clinical suspicion) and 5 were laboratory confirmed (positive PCR or electron microscopy results). Demographic and health data were not available for 7 animals with presumptive sealpox; thus, these animals were not included in subsequent analyses, leaving a total of 23 infected pinnipeds at 9 centers for analysis of characteristics associated with sealpox virus infection. Individual animals from 4 species were identified as having sealpox: California sea lions (8), harbor seals (11), gray seals (3), and northern elephant seals (1) (Table 2). Sealpox was diagnosed upon arrival at a center for 5 (22%) animals (all wild), <5 weeks after arrival for 11 (48%) animals, and >5 weeks after arrival for 2 (9%) animals (Table 2). The timing of illness onset was independent of the animal’s geographic location, species, and age. Most (83%) sealpox was diagnosed in pups, but the prevalence of infection was equivalent for adults and pups (1.2% and 1.8%, respectively).

**Table 2:** Outcome of sealpox infection among 23 pinnipeds at 9 marine mammal rehabilitation facilities, North America, 2007–2009

Characteristic	Outcome of infection, no. animals			Total no. animals
	Illness resolved	Animal died or was euthanized	Unknown	
Species				
California sea lion	6	2	0	8
Gray seal	2	1	0	3
Northern elephant seal	0	1	0	1
Harbor seal	8	3	0	11
Male				
Pup, <1 y of age	9	3	1	13
Adult	0	1	0	1
Female				
Pup	6	1	0	7
Adult	0	2	0	2
Time of lesion development				
At admission to facility	1	3	0	4
<5 wk after admission	9	2	0	11
>5 wk after admission	2	0	0	2
Health status				
Diseased	0	4	0	4
Malnourished	8	1	0	9
Injured plus malnourished or diseased	9	1	0	10

Of the ill animals, 9 (39%) were malnourished, 4 (17%) had concurrent illness, and 10 (44%) had injuries and were malnourished or had concurrent illness (Table 2). Those with concurrent illness died or were euthanized (including all adults in which sealpox was diagnosed), while most animals with malnutrition recovered (Table 2). Informants from 5 marine centers reported transmission of sealpox from infected animals to unhealthy convalescing pinnipeds at their facility, 4 reported transmissions to healthy pinnipeds, and 6 reported transmission among pups. Of the 9 facilities that housed infected animals, 4 reported isolating them to prevent transmission.

To identify factors that might be associated with transmission of sealpox virus in marine mammal facilities, we obtained information about disinfectant use in pens, the number of animals housed per pen, and other housing characteristics (Table 3). Individual facilities had 1 to >16 enclosures and housed <83 pinnipeds during the 12 months before completing the survey. All facilities used at least 1 type of disinfectant, and all reported cleaning pens at least 1× each week (Table 3). To assess whether medical screening and treatment practices might affect detection and prevention of sealpox, we obtained information about facility admission protocols for pinnipeds as well as laboratory and medical resources available at the facility. All facilities had quarantine or isolation enclosures for sick or newly admitted animals, and all facilities included a physical examination as part of their standard protocol for pinnipeds entering the facility. Of the 9 facilities that had animals with sealpox, 9 (100%) reported isolating newly admitted pinnipeds and collecting blood samples for pathogen screening; 4 (44%) also did fecal flotation and culture; and 2 (22%) did other tests, including, including urinalysis and radiography or ultrasound (Table 3). All facilities had veterinarians on site and used third-party diagnostic laboratories; 64% also had a veterinary diagnostic laboratory on site.

**Table 3:** Characteristics of 11 marine mammal rehabilitation facilities that did or did not report sealpox among pinnipeds during the 12 months before survey of marine mammal centers, North America, 2007–2009

Characteristic	Sealpox reported in facility in past 12 mo	
	No	Yes
Animal enclosures		
Shared by adults and pups*	1	2
Shared by different species	2	5
Quarantine space available	2	9
Frequency of pen disinfection		
2×/d	0	3
1×/d	0	6
<1×/wk	2	0
Disinfectant used†		
Any use	2	9
Bleach	1	8
Virkon	1	3
Chlorhex	2	2
Iodophores	0	2
Standard protocols for newly admitted pinnipeds†		
Isolation/quarantine	1	9
Physical examination	2	9
Blood tests‡	1	9
Vaccination§	1	1
Coast		
Atlantic	1	4
Pacific	1	5
Enclosure material†		
Plastic	1	4
Fiberglass	1	7
Cement	1	5
Epoxy resin	1	1
Wire	0	1

*Adults, >1 y of age; pups, <1 y of age. †Responses were not mutually exclusive. ‡Tests performed included pathogen screening for morbillivirus, Brucella spp., rabies virus, Leptospira spp., West Nile virus, heartworms, intestinal helminths, and avian influenza virus. §Vaccinations included those against West Nile virus, rabies virus, and Leptospira spp.

Overall, no significant association was found between sealpox infection and species, sex, or age in the study population (Table 2), and no significant associations were found at the facility level. The likelihood of a pinniped having sealpox was greater in facilities on the Atlantic coast (4% of animals) than the Pacific coast (1% of animals) (odds ratio 3.53; p = 0.002). However, this observation mainly results from case clusters at 2 facilities on the Atlantic coast. All of the ill animals were characterized as having been rescued or having been in rehabilitation (Table 1). All but 1 facility reported housing adults and pups together and housing different pinniped species together; there was no association with sealpox infection (Table 3). Of the 11 surveyed facilities, 9 (82%) cleaned the enclosures at least daily with bleach-based cleaning products (Table 3). No significant associations were found between type of disinfectant used and odds of sealpox infection. All facilities that housed infected animals reported cleaning enclosures at least daily.

None of the facility informants reported cases of sealpox infections among humans at the facility during the prior 12 months, but informants at 2 facilities reported cases of physician-diagnosed sealpox among workers in the previous 10 years. At both facilities, the affected persons were reported to have handled live animals with skin or oral lesions or both. Use of personal protective equipment (PPE) was reported at all facilities: 100% of respondents reported use of gloves; 91%, rain pants, overalls, or suits; and 9%, goggles and masks.

In addition to the survey of marine mammal rehabilitation facilities, we asked marine mammal workers attending a conference about their knowledge and experience with sealpox. Thirty-one conference attendees completed the survey. Of the 31 respondents, 17 (55%) reported observing pinnipeds affected with sealpox virus in rehabilitation facilities or in the wild, of which 9 were in North America, 5 in northern Europe, and 2 in New Zealand (1 location not specified). (In the United States, human sealpox virus infection is not a nationally reportable condition. However, as a rare infection, it would be considered notifiable in most states.) Of the 17 workers, 13 reported knowledge that sealpox virus can infect humans, and most (15/17) reported that they had learned this information from colleagues or educational material distributed at their workplace. Of the 31 respondents, 7 (23%) reported they had seen or known about a human case of sealpox: 2 reported that a colleague had received a diagnosis of sealpox, and 5 reported that they themselves had received a diagnosis of sealpox at some point during the past 15 years. Two of these 7 persons stated that their physician had prescribed tetracyclines to treat their infections. Tetracyclines are ineffective against sealpox infections; however, antimicrobial drugs are effective against a mycoplasma-associated infection, called seal finger, which is clinically similar to and can be confused with sealpox.

## Discussion

For sea lions entering rehabilitation facilities, a history of rehabilitation is a strong indicator of the risk for poxvirus lesions (*14*). In our survey, facility informants reported that 2% of the animals housed in the marine mammal facilities surveyed had laboratory-confirmed or presumptive sealpox during the preceding 12 months. The proportion of infected animals was higher in facilities on the Atlantic than Pacific coast; however, on balance, more animals were housed in rehabilitation facilities on the Pacific coast. California sea lions accounted for the majority of pinnipeds under observation and for the majority of infected animals. Most infected animals were pups and were male, a finding consistent with previous observations (*1**,**14*). Documented infections have typically been reported for young animals brought into rehabilitation environments, which may reflect increased susceptibility among juvenile animals, particularly those under stress (*2*). Malnutrition, a hallmark of rescued pups, seemed not to indicate a poor prognosis, whereas injury, older age, and concurrent illness did.

Approximately one fifth of infected animals arrived at the rehabilitation facility with symptoms of sealpox infection, and infection was observed within 5 weeks after arrival for half of the animals. We did not attempt to address whether underlying poor health status predisposes an animal to infection with sealpox virus or vice versa. However, 5 facility informants reported sealpox transmission in their facility to both unhealthy and healthy pinnipeds, suggesting that, in captivity, the disease can develop even in apparently healthy animals. The transmission dynamics of sealpox virus have not been well described, but transmission between pinnipeds is likely to occur during suckling; through other forms of skin-to-skin contact; or by fomites, which are of particular concern for captive animals. Further studies are needed to explain transmission dynamics.

Serosurveys suggest that sealpox virus is persistently infecting most wild California sea lions, and other species of pinnepeds may also be persistently infected with other sealpox species (*23*). Thus, for persons who rehabilitate animals, risks for zoonotic transmission of these agents might be appreciable. Vigilant adherence to quarantine and institutional hygiene practices, including the use of PPE by staff, likely serves to diminish risks for virus transmission to humans and may explain why reports of human infection with sealpox virus are rare (*16**,**17*). In our study, all marine mammal workers who knew that sealpox virus is zoonotic reported that they had learned this information from colleagues and educational material provided by their workplace, and all facilities reported that PPE (gloves, boots) were made available to all workers; however, some marine mammal workers indicated that PPE is rarely used when pinnipeds are handled in the wild. Sealpox is only 1 of many potential hazards associated with wildlife rehabilitation activities; workplace education programs should highlight the risk for zoonotic disease transmission from pinnipeds to marine mammal workers and the use of PPE when handling animals in the wild.
